# The challenge in treating locally recurrent T3-4 nasopharyngeal carcinoma: the survival benefit and severe late toxicities of re-irradiation with intensity-modulated radiotherapy

**DOI:** 10.18632/oncotarget.15896

**Published:** 2017-03-04

**Authors:** Yun-Ming Tian, Wei-Zeng Huang, Xia Yuan, Li Bai, Chong Zhao, Fei Han

**Affiliations:** ^1^ Department of Radiation Oncology, Hui Zhou Municipal Centre Hospital, Huizhou, Guangdong, P.R. China; ^2^ Department of Radiation Oncology, Sun Yat-Sen University Cancer Centre, State Key Laboratory of Oncology in South China, Guangzhou, Guangdong, P.R. China; ^3^ Department of Medical Oncology, Hui Zhou Municipal Centre Hospital, Huizhou, Guangdong, P.R. China

**Keywords:** nasopharyngeal carcinoma, local recurrence, intensity-modulated radiotherapy, late complications

## Abstract

**Background:**

Effective treatments for patients with advanced locally recurrent nasopharyngeal carcinoma (NPC) are limited. This investigation was to determine the potential benefits from re-irradiation by intensity-modulated radiotherapy (IMRT) on survival and the effects of severe late toxicities.

**Methods:**

A retrospective study was conducted in 245 patients diagnosed with locally recurrent T3–T4 NPC who had undergone re-irradiation with IMRT. Follow-up data was colleted and factors associated with survival and severe late toxicities were analyzed.

**Results:**

The 5-year local-regional failure-free survival, distant failure-free survival and overall survival rates were 60.9%, 78.3% and 27.5%, respectively. The presence of severe late complications, recurrent T4 disease and gross tumor volume >30 cm^3^ were associated with poor survival. The incidences of mucosal necrosis, temporal lobe necrosis, cranial neuropathy and trismus were 22.0%, 14.6%, 27.0% and 14.6% respectively. Conclusions: Re-irradiation with IMRT is an effective choice in patients with locally recurrent T3–T4 NPC. However, the survival benefits can be partly offset by severe late complications and optimum treatments in these patients remain a challenge.

## INTRODUCTION

Nasopharyngeal carcinoma (NPC) is sensitive to radiotherapy and chemotherapy. Although excellent disease control rates have been achieved in patients with NPC after comprehensive treatments, local recurrence remains a major cause of treatment failure, particularly in patients with advanced disease [1–3]. Salvage treatments with curative intent have improved long-term survival in selected patients with locally recurrent NPC. These mainly involve aggressive surgery or re-irradiation [4]. Successful outcomes have been reported following salvage surgical resection and brachytherapy in cases of limited disease or localized in the nasopharynx [5, 6]. Stereotactic radiotherapy (SRT) proved effective in cases involving relatively small tumor volumes (diameter ≤3 cm) [7–9]. However, the majority of locally recurrent NPC were presented with extensive invasion, limiting the choice of effective salvage treatments. Re-irradiation with external beam radiotherapy remains the principle modality. Conventional radiotherapy is often difficult and results in poor survival and high incidence of severe late complications due to the impact of high dose primary radiation on critical organs located in the vicinity of the nasopharynx [10, 11]. In contrast, intensity-modulated radiotherapy (IMRT) offers a more favorable balance between target coverage and the sparing of adjacent organs [12, 13]. IMRT has proved feasible in cases of locally recurrent NPC; therefore, the aim of this study was to assess the long-term survival benefits and late radiation-induced toxicities in locally recurrent T3-T4 NPC who had undergone re-irradiation with IMRT.

## RESULTS

### Patient characteristics

Of the 245 patients selected for this study, 196 were male (80.0%) and 49 were female (20.0%). The median age was 46 years (range: 21-69 years). They included 118 patients (48.2%) with T3 disease and 127 patients (51.8%) with T4 disease. Severe late complications after the first course of radiotherapy were presented in 47 patients (19.1%) including 16 with trismus, 13 with mucosa necrosis, 11 with radiation-induced encephalopathy, 5 with cranial nerve palsy, and 2 with both trismus and radiation-induced encephalopathy. The clinical characteristics of the patients are summarized in Table 1.

**Table 1:** Clinical characteristics of 245 patients with locally recurrent nasopharyngeal carcinoma

Characteristic	No. of patients (%)
Gender Male female	49 (20.0) 196 (80.0)
Age (years) Median Range Prior radiotherapy technique (RT) Conventional RT Conformal RT IMRT	46 21–69 228 (93.1) 8 (3.3) 9 (3.7)
Presence of severe late complications No Yes	198 (80.8) 47 (19.2)
Disease-free interval (months)^1^ 6-12 13-18 19-24 >24	38 (15.5) 30 (12.2) 45(18.3) 132 (53.8)
Recurrent T classification T3 T4	118 (48.2%) 127 (51.8%)
Presence of nodal recurrence No Yes	208 (84.9) 37 (15.1)
GTV-nx (cm3) ≤30 >30	72 (29.3) 173 (70.7)
Mean dose of GTV-nx (Gy) Median Range ≤68 Gy >68 Gy	70 60.1-78.7 93 (37.9%) 152 (62.1%)
Chemotherapy Yes No	157 (64.1) 88 (35.9)

^1^ Disease-free interval was defined from the date of completion of treatment to diagnosis of recurrence or final follow-up if sooner; GTV-nx, gross tumor volume in the nasopharynx.

### Survival outcomes and causes of death

The median follow-up time was 24.0 months (range from 2 to 132 months). The 5-year local-regional failure-free survival (LRFFS)and distant failure-free survival (DFFS) and overall survival rates (OS) were 60.9%, 78.3% and 27.5% respectively. Local-regional failure after completion of IMRT was confirmed in 79 (32.2%) patients. They comprised of 74 patients with local failure sited solely in the nasopharynx, 4 with regional failure sited in the lymphatic region of the neck, and one patient with local-regional failure. Distant metastasis was confirmed in 33 (13.5%) patients, including 13 with bone metastasis, 10 with lung metastasis, 5 with liver metastasis and 5 with multi-organ metastases.

A total of 184 (75.1%) patients died during follow-up. Of these, 91 were associated with disease progression (including 65 from local-regional failure, 19 from distant metastasis and 7 from both); 72 were caused by radiation-induced injuries including 33 from massive hemorrhaging or mucosa necrosis, 16 from radiation encephalopathy and 23 from radiation-associated trismus or cranial IX-XII neuropathy (including 8 patients with malnutrition, 11 aspiration pneumonia and 4 patients with both of them). A further 10 patients died from a known medical condition (e.g. cardiac disease, digestive diseases and leukemia) and 11 died from an unknown cause.

### Treatment-related complications

Almost all of the patients developed mild to moderately acute complications during radiotherapy, including mucositis and xerostomia. Severe acute complications were less common, with only 31 (12.7%) patients developing grade III acute mucositis, and 9 (3.7%) with synchronous nodal recurrence who developed grade III skin injuries. The severity and incidence of xerostomia were not recorded in this study due to the difficulty in evaluation.

Late complications ≥grade III recorded and scored during the follow-up period are summarized in Table-2. Mucosal necrosis was the most common complication and was observed in 66 (26.9%) patients, including 39 (15.9%) cases involving the internal carotid artery or other vessels eventually leading to hemorrhage. The incidence of mucosal necrosis was significantly higher in patients with a disease-free interval between primary and re-irradiation (DFI) ≤24 months compared to those with a DFI >24 months (31.2% vs. 19.5%; χ^2^ = 4.56; *P* = 0.05) or a gross tumor volume of nasopharynx (GTV-nx) >30 cm^3^ compared to those with a GTV-nx ≤30 cm^3^ (30.8% vs. 12.6%; χ^2^ = 8.34; *P* < 0.01). Furthermore, patients with a GTV-nx >30 cm^3^ were more prone to hemorrhage (21.2% vs. 10.4%; χ^2^ = 6.32; *P* = 0.04).

**Table 2:** Grade ≥III complications in 245 patients of rT3-4 NPC

Complication	No. of patients (%)	Time to Occurrence(month)
Mucosal necrosis	66 (26.9%)	5.0 (1-57)
Hemorrhage	39 (15.9%)	10.0 (1-66)
Temporal lobe necrosis	54 (22.0%)	14.0 (2-96)
Cranial nerve palsy	35 (14.3%)	12.0(2-72)
Trismus (≤ 1 cm)	35 (14.3%)	7.0(2-72)
Hearing loss	11 (4.5%)	15.0(3-36)

**Figure 1:**
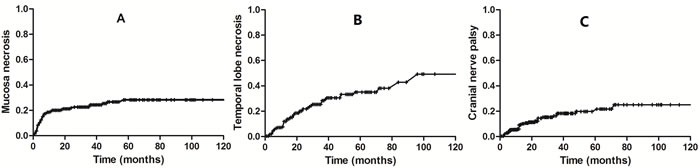
The cumulative hazard risk of the occurrence of mucosal necrosis (**A**), temporal lobe necrosis (**B**) and cranial neuropathy (**C**) during a long-term follow-up period.

Temporal lobe necrosis was identified in 54 (22.0%) patients, leading to cranial neuropathy in 35 (14.3%) cases, trismus in 35 cases (14.3%) and hearing loss in 11 (4.5%) cases (The results were summarized in the tab). The incidence of temporal lobe necrosis was significantly higher in patients with stage T4 disease or DFI≤24 months when compared to those with stage T3 disease (27.6% vs. 17.2%; χ^2^ = 5.68; *P* = 0.03) and DFI >24 months (26.1%vs. 16.5%; χ^2^ = 8.15; *P* < 0.01). Furthermore, the patients with DFI≤24 months had higher incidence ofcranial neuropathy when compared with DFI >24 months (19.7% vs. 8.0%, χ^2^ = 11.12; *P* =0.03). However, no factor was demonstrated associated with the incidence of trismus. The cumulative hazard risk of the occurrence of the main severe late complications were shown by the Figure 1.

### Prognostic factors

Potential prognostic factors included clinical characteristics (age, gender and presence of severe radiation-induced complications), disease status (recurrent T classification, synchronous nodal recurrence, GTV-nx and DFI) and treatment modality (chemotherapy and re-irradiation doses).

Univariate analysis identified age ≤50 years, the presence of severe late complications, recurrent T4 disease, GTV-nx >30 cm^3^ and synchronous nodal recurrence as significant adverse prognostic factors for OS. Recurrent T4 disease was also associated with poor LRFFS; however, none of these factors were significantly associated with DFFS.

**Figure 2:**
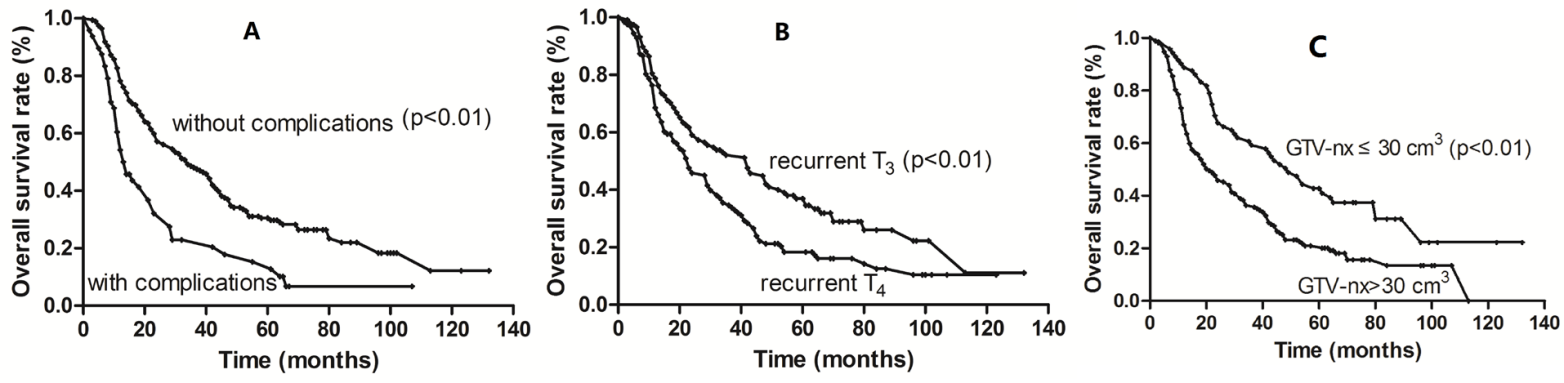
Kaplan-Meier survival curves according to the presence of significant complications (**A**), recurrent T stage (**B**) and tumor volume (**C**).

Multivariate analysis identified the presence of severe late complications, recurrent T4 disease and GTV-nx >30 cm^3^ as independent prognostic factors of poor survival. The 5-year OS of patients who presented with severe late complications was significantly poorer than those without complications (15.3%, vs. 30.5%; *P* < 0.01). The 5-year OS was significantly poorer in patients with T4 disease compared to those with T3 disease (18.4% vs. 36.9%; *P* < 0.01) and patients with GTV-nx >30 cm^3^ compared to those with GTV-nx ≤30 cm^3^ (43.4% vs. 20.9%; *P* < 0.01). Recurrent T4 disease was also associated with worse LRFFS than T3 disease (54.2% vs. 68.3%; *P* = 0.03). The survival curves are shown in Figure 2 and the results are summarized in Table 3.

**Table 3:** Variables associated with overall survival in 245 patients with locally recurrent nasopharyngeal carcinoma

Characteristic	5-year OS (%)	Univariate analysis		Multivariate analysis	
		HR (95% CI)	*P*-value	HR (95% CI)	*P*-value
Gender, male/female	27.0 (29.2)	0.89 (0.62–1.22)	0.48	0.96 (0.65–1.41)	0.84
Age (years), ≤50/>50	31.6 (22.0)	1.33 (1.01–1.87)	0.04	1.20 (0.88–1.63)	0.15
Significant complications, no/yes	30.5 (15.3)	2.01 (1.40–2.82)	<0.01^a^	2.13 (1.48–3.07)	<0.01^a^
Disease-free interval (months)^1^ >24 19-24 13-18 6-12	27.0 28.7 44.4 13.2	1.12 (0.57–2.61) Baseline 1.04 (0.71–1.54) 1.62 (0.95–2.75) 0.68 (0.46–1.08)	0.63 - 0.80 0.07 0.06	1.09 (0.80–1.45) Baseline 1.16 (0.78–1.74) 1.32 (0.78-2.25) 0.53 (0.35–0.80)	0.60 0.45 0.29 0.03
Recurrent T classification, rT3/T4	36.9 (18.4)	1.57 (1.17–2.14)	0.03^a^	1.68 (1.27–2.33)	<0.01^a^
Synchronous nodal recurrence, no/yes	28.7 (21.3)	1.30 (0.87–1.97)	0.19	1.53 (0.91–2.34)	0.09
Volume of GTV-nx (cm^3^), ≤30/>30	43.4 (20.9)	1.96 (1.40–2.75)	0.01^a^	1.78 (1.24–2.53)	<0.01^a^
Chemotherapy, no / yes	24.2 (29.5)	1.21 (0.76–2.39)	0.37	1.08 (0.87–1.46)	0.12
Re-irradiation dose (Gy), ≤68/>68	28.1 (26.8)	1.18 (0.87–1.60)	0.64	1.25 (0.91–1.47)	0.33

^a^ Statistical significance.

^1^ CI (confidence interval); HR (hazard ratio); OS (overall survival).

## DISCUSSION

The majority of patients with recurrent NPC were presented with extensive invasion and a high risk of infiltration. Currently, effective salvage treatments in these patients are limited. In a study of 90 patients with recurrent or residual NPC, SRT was found to be superior to conventional radiotherapy, achieving a 3-year local control rate of 75.0% and a disease-specific survival rate of 57.2%. This was attributed to the delivery of a higher dose of radiation [9]. However SRT is not commonly applied due a lack of expertise and the selection of suitable patients. In comparison, a study of two-dimensional radiotherapy in 654 patients with locally recurrent NPC only achieved a 5-year local control of 11% in those patients with recurrent T3-T4 disease, and the cumulative incidence of major late complications post-treatment was 26% [10]. The superior dose distribution and sparing of normal tissue suggests that IMRT may be the most effective curative treatment in patients with advanced NPC who exhibit extensive invasion or large tumor volumes [12, 13]. Our study supported this suggestion, by demonstrating that patients with recurrent T3-T4 NPC achieved 5-year LRFFS and OS rates of 60.9% and 27.5%, respectively, after receiving re-irradiation with IMRT. Consistent with previous studies [14, 15], our analyses also identified recurrent T-classification and tumor volume as the most significant prognostic factors in patients with locally recurrent NPC. This may have been due to decreased radiation sensitivity caused by poor blood supply and hypoxia, a suboptimal dose distribution resulting from protection of critical structures, or an increase in the incidence of severe late complications caused by larger levels of high-dose radiation.

Severe late complications are a major cause of death in patients with recurrent NPC after high-dose re-irradiation. Mucosal necrosis is considered the most common late complicationand with no accepted effective treatment [16]. It is believed to be related to tissue injury and chronic non-healing wounds caused by hypoxia, hypovascularity and hypocellularity after irradiation [17]. Mucosal necrosis accompanied by severe headache and foul odor can severely reduce the patient's quality of life and can result in fatal hemorrhage if the internal carotid artery are involved. The incidence of mucosal hemorrhage in our study population was 27.0%; approximately 45.0% of these cases eventually extended to the internal carotid artery resulting in fatal massive hemorrhage. The incidence of mucosal necrosis was higher in patients with a DFI ≤24 months or GTV-nx >30 cm^3^ compared to those with a DFI >24 months or GTV-nx ≤30 cm^3^.

Temporal lobe necrosis and cranial neuropathy are debilitating and potentially fatal complications in patients with locally recurrent NPC following re-irradiation. A previous study involving 227 patients with locally recurrent NPC found that 71 patients developed temporal lobe necrosis after receiving two courses of radiotherapy. The interval between the two courses and the summation of the maximum doses were identified as independent factors [18]. Consistent with this study, our results showed patients with a DFI ≤24 months were more prone to developing temporal lobe necrosis than those with DFI >24 months. The incidence of temporal lobe necrosis was also higher in patients with recurrent T4 disease compared to those with T3 disease, due to the potential higher radiation dose for the temporal lobe. Cranial neuropathy was also common after re-irradiation with the incidence of 14.3%, leading to malnutrition by the difficulty in swallowing and aspiration pneumonia. The factor of DFI was associated with the incidence of cranial neuropathy.

Together, these findings suggested that the risk of severe late complications may be minimized by delivering the optimal total radiation dose and fraction size. There are several studies in support of this supposition: A phase II, randomized trial studied the effects of total dose and fraction size on survival in patients with locally recurrent NPC following treatment with IMRT. The results revealed that the incidences of mucosal necrosis and massive hemorrhage were significantly higher in patients receiving 68Gy/34fs than those receiving 60Gy/27fs [19]. A retrospective study comparing the use of SRT and conformal radiotherapy (CRT) for re-irradiation showed that the incidence of severe late toxicities with SRT was significantly lower than with CRT (21% vs. 48%, respectively) [20]. However, the optimal radiation dose and fraction size was still uncertain to balance the local control and the radiation-induced injuries.

Retrospective studies involving small sample sizes have indicated that combining radiotherapy with chemotherapy, such as induction chemotherapy, could facilitate target coverage by shrinking the tumor in cases of advanced and extensive local recurrence. A 1-year local control rate of 75% was reported in the treatment of patients with recurrent T4 disease with intracranial extension by employing this technique prior to re-irradiation with IMRT [21]. An earlier study related to concurrent chemotherapy with cisplatin followed by consolidation cisplatin and 5-Fu reported a 1-year progression-free rate of 42% [22]. These findings suggest that a combined modality approach may improve the outcome of patients with locally recurrent NPC; however, evidence from prospective phase III trials will be needed to verify this proposition.

This study demonstrates that re-irradiation with IMRT is an effective choice for patients with locally recurrent T3-T4 NPC; however, the benefits are often offset by severe late complications and this is presenting a considerable challenge in the treatment of these patients. Further research is required to determine the optimum balance between disease control and protection of critical organs.

## MATERIALS AND METHODS

### Patient selection

A total of 245 patients were diagnosed with locally recurrent T3-T4 NPC and underwent re-irradiation with IMRT between January 2001 and December 2010 in the Sun Yat-sen University Cancer Center and Huizhou Municipal Centre Hospital. Local recurrence was histologically confirmed in 203 patients. Deep-seated recurrences in the skull base and intracranial space were confirmed by radiological examinations and clinical symptoms in the remaining 42 patients. Patients selected for re-irradiation by IMRT met the following criteria: (1) no evidence of distant metastases at diagnosis; (2) an interval >6 months between the end of primary radiotherapy and the diagnosis of recurrence; (3) a Karnofsky performance status score ≥70.

Pretreatment evaluation consisted of a complete medical history and physical examination, renal and liver function tests and an electrocardiogram. Magnetic resonance imaging (MRI) of the nasopharynx and neck, chest X-ray, abdomen sonography and bone scans were administrated. Positron emission tomography (PET) was performed if indicated by preceding examinations. Imaging data was used to restage patients according to the 2002 American Joint Committee on Cancer classifications.

The study was approved by the Ethical Review Committee of Sun Yat-Sen University Cancer Centre. Written consent was given by the patients before receiving the treatments..

### Intensity-modulated radiation therapy

Accurate delineation of target volumes and organs at risk is essential in radiotherapy planning and is a basic requirement for improving outcome and quality of life in patients undergoing treatment. This was based on CT simulations and the following parameters were evaluated by a clinician according to the International Commission on Radiation Units and Measurements (ICRU) reports 50 and 62: gross tumor volume (GTV) was defined as gross disease in the nasopharynx (GTV-nx) and neck (GTV-nd); the clinical target volume (CTV) was defined as the GTV plus a 1.0-1.5 cm margin. A smaller margin (<3 mm) was included if the tumor was located adjacent to critical intracranial structures. These included the brainstem, spinal cord, optic nerves, optic chiasm, temporal lobes, pituitary gland, parotid glands, temporomandibular joints and mandible. The planning target volume (PTV) consisted of the CTV plus an additional 2-3 mm margin to compensate for set-up errors.

All patients received a full course of IMRT with 6MV X-rays generated by a Clinac-600C linear accelerator (Varian Medical Systems, Palo Alto, CA, USA). The patient's head and neck were immobilized using a thermoplastic mask. The prescribed doses were 60-70 Gy to the GTV-nx and 50-54 Gy to the CTV, delivered in 27-35 fractions. The medians of the minimum dose, mean dose and maximum dose to the GTV-nx were 56.0 Gy (range, 33.3-70.1 Gy), 70.0 Gy (range, 60.1-78.7 Gy) and 75.7 Gy (range, 65.0-85.1 Gy), respectively (The dose of tumor target is summarized in Table 1).

The dose constraints of the critical normal structures were based on the threshold doses to the normal structures and the disease-free interval (DFI) after completion of primary radiotherapy to the date of recurrence.

### Chemotherapy

Cisplatin-based induction or concurrent chemotherapy was administrated in patients with bulky gross tumors or with a relatively short DFI (~ 6 months) between the end of primary radiotherapy and recurrence. A total of 157 (64.1%) patients received comprehensive treatment: 54 patients received concurrent chemotherapy alone; 65 patients received induction chemotherapy alone; and 38 patients received both induction and concurrent chemotherapy. The regimen for induction chemotherapy was based on the pretreatment evaluation and included TPF (paclitaxel+ cisplatin+5-fluorouracil), TP (paclitaxel+ cisplatin) and PF (cisplatin+5-fluorouracil). Concurrent chemotherapy was based on cisplatin alone.

### Follow-up and statistical analysis

During treatment, all patients were examined weekly and acute radiation toxicities were recorded. After completion of treatment, patients were evaluated at least once every three months during the first three years and every six months thereafter. A complete clinical history and examination, MRI of the head and neck, chest X-ray radiography and abdominal ultrasound were performed annually. Late radiation-induced toxicities were recorded and scored according to the radiation morbidity scoring criteria of the Radiation Therapy Oncology Group (RTOG). The severity of late radiation-induced complications were classed as severe (Grade III-IV) or fatal (Grade V). Mucosal necrosis, temporal lobe necrosis, trismus with a dental gap ≤1 cm, cranial neuropathy and ear symptoms were evaluated and analyzed. The date of the last follow-up was 30^th^ June 2015.

Overall survival, local-regional failure-free survivaland distant failure-free survival were calculated from the date of the completion of IMRT to the date of each event or the last follow-up. Calculations were performed by the Kaplan-Meier method. Log-rank tests were used to determine differences in survival between different prognosticators. Multivariable analysis was performed using the Cox proportion hazards model. The categorical variables, including severe late complications, were compared using the χ^2^ test. A value of *P* < 0.05 was considered significant.
